# Effective Adsorption and Sensitive Detection of Cr(VI) by Chitosan/Cellulose Nanocrystals Grafted with Carbon Dots Composite Hydrogel

**DOI:** 10.3390/polym13213788

**Published:** 2021-11-01

**Authors:** Hua Zeng, Zhiyuan Hu, Chang Peng, Lei Deng, Suchun Liu

**Affiliations:** 1 College of Food Science and Technology, Hunan Agricultural University, Changsha 410128, China; zh@hunau.edu.cn (H.Z.); huzhiyuan@hncu.edu.cn (Z.H.); 2 School of Chemistry and Materials Science, Hunan Agricultural University, Changsha 410128, China; pengchang@hunau.edu.cn (C.P.); leideng@hunau.edu.cn (L.D.)

**Keywords:** chitosan, cellulose nanocrystals, carbon dots, adsorption, hexavalent chromium

## Abstract

Due to its lethal effect on the human body and other creatures, Cr(VI) ions have attained widespread public attention, and an effective adsorbent for removing Cr(VI) ions is vital. Chitosan (CS)/cellulose nanocrystals grafted with carbon dots (CNCD) composite hydrogel with strong sorption ability and sensitive detection ability for Cr(VI) was formed. The cellulose nanocrystals (CN) offered a natural skeleton for assembling 3D porous structures, and then improved the sorption ability for Cr(VI); moreover, carbon dots (CD) acted as a fluorescent probe for Cr(VI) and provided Cr(VI) adsorption sites. With a maximum adsorption capacity of 217.8 mg/g, the CS/CNCD composite hydrogel exhibited efficient adsorption properties. Meanwhile, with a detection limit of 0.04 μg/L, this hydrogel was used for selective and quantitative detection of Cr(VI). The determination of Cr(VI) was based on the inner filter effect (IFE) and static quenching. This hydrogel retained its effective adsorption ability even after four repeated regenerations. Furthermore, the economic feasibility of the CS/CNCD composite hydrogel over activated carbon was confirmed using cost analysis. This study provided one new method for producing low-cost adsorbents with effective sorption and sensitive detection for Cr(VI).

## 1. Introduction

Due to rapid industrial development, water contamination by heavy metals has become more and more serious [1]. To remove heavy metals, many traditional methods, such as chemical precipitation, membrane filtration, photocatalytic degradation, and adsorption are currently used [2]. Adsorption is regarded as one of the most promising approaches in these methods. As a result, creating an efficient adsorbent is essential.

CS has received widespread attention as an adsorbent for heavy metal ions due to its low cost and great adsorption potentials [3]. CS is blended with other materials leading to improved sorption performance. Pavithra et al. formed a surface-tailored chitosan/orange peel composite hydrogel to remove Cr(VI) and Cu(II) ions from synthetic wastewater, and then measured the adsorption capacity for these two ions [4]. Hao et al. prepared N-carboxymethyl chitosan hydrogel, analyzed its morphology and structure, studied the influencing factors of adsorption performance, and discussed the intrinsic mechanism of the adsorption process [5]. The adsorption thermodynamics, kinetics, and adsorption mechanism of a composite chitosan-glucose hydrogel for sorption of Co^2+^ ion were discussed, and the adsorption capacity of 202 mg/g for Co^2+^ was demonstrated [6]. Although researchers have made great efforts, improving the adsorption capacity for heavy metals by the chitosan-based material is still a challenge. A general solution would be to introduce nanoparticles with excellent properties in order to increase adsorption capacity [7]. In this regard, we propose introducing cellulose nanocrystals (CN). Firstly, CN can serve as a scaffold around which a network structure can form. Second, CN can provide a large number of hydroxyl and carbonyl functional groups as heavy metal sorption sites [8]. Therefore, CS/CN composite hydrogel has great potentiality in the development of novel adsorbents.

Carbon-based fluorescent nanomaterials, such as carbon dots (CD), have recently stimulated the interest of researchers due to their unique optical and nontoxic properties [9,10]. Many studies have shown that CDs can be used to detect Cr(VI) optically [11,12]. However, some issues remain in sensor probe application, such as CD agglomeration with increasing detection and storage time [13]. One possible solution to overcome this problem is to graft with other materials, such as CN [14].

In this article, we aimed to create chitosan/CN grafted with CD (CS/CNCD) composite hydrogel for Cr(VI) detection and sorption. This hydrogel’s morphology, structure, fluorescence properties, adsorption, and detection characteristics were investigated. In addition, the sorption interaction between Cr(VI) and this hydrogel was confirmed. The economic analysis of this hydrogel was also explored.

## 2. Materials and Methods

### 2.1. Materials

CS with a deacetylation degree of ca. 90% (J&K Scientific Ltd., Beijing, China). 2,2,6,6-Tetramethylpiperidinooxy (TEMPO) (98%, Aladdin, Shanghai, China), 1-(3-Dimethylaminopropyl)-3-ethylcarbodiimide hydrochloride (EDC) (98%, Aladdin, Shanghai, China), Citric acid (99.8%, Aladdin, Shanghai, China), Ethylenediamine (98%, Aladdin, Shanghai, China), N-Hydroxysuccinimide (NHS) (98%, Aladdin, Shanghai, China), and Glutaraldehyde (GD) (50% in H_2_O, Aladdin, Shanghai, China).

### 2.2. Synthesis of CN

As previously reported, the CN was produced using never-dried bleached wood pulp [15]. Bleached Wood pulp (Jin Yi Inc., Shanghai, China) (2.0 g), NaBr (Aladdin, Shanghai, China) (0.2 g), and 2,2,6,6-Tetramethylpiperidinooxy (TEMPO, 0.04 g) were mixed in water (200 g). The mixture was then thoroughly mixed with an aqueous solution of NaClO (Aladdin, Shanghai, China) (6 mL, 5% chlorine solution). The pH was maintained at 10, and this oxidation lasted for 24 h. Subsequently, the mixture was washed with water. Six passes through a microfluidizer (M110P, Microfluidics Corp., Newton, MA, USA) were made with the obtained mixture. Subsequently, the CN suspension was prepared. 

### 2.3. Synthesis of CD

The water-dispersible CD was synthesized and purified by Zhu et al. [16]. Citric acid (2.1 g) and Ethylenediamine (268 μL) were dissolved in water (20 g). The mixture was placed in a Teflon reaction vessel (Yi Chuang company, Xi’an, China), sealed, and autoclaved for 5 h at 300 °C. The autoclave was then cooled. To remove excess reactants, the solution was dialyzed in a dialysis bag (500–1000 D) (Fisher Scientific, Pittsburgh, PA, USA) against water for three days. The CD could be obtained within this dialysis bag. High-resolution TEM images of the CD are provided in Figure S1. 

### 2.4. Synthesis of CNCD

The CN suspension (10 g, 1.0 wt%) was activated with 1-(3-Dimethylaminopropyl)-3-ethylcarbodiimide hydrochloride (EDC, 20 mg) and N-Hydroxysuccinimide (NHS, 16 mg) under vigorous stirring at pH 5 for 15 min. Further, a CD solution (10 mL, pH 9.2, phosphate buffer (Aladdin, Shanghai, China)) containing various mass fractions (0.02 wt%, 0.04 wt%, 0.06 wt%, 0.08 wt%, 0.10 wt%, and 0.12 wt%) was added. For 12 h, the coupling reaction was carried out with mild agitation at 25 °C. The resulting suspension was washed and dialyzed against water. Since the mass ratio of CN and CD could affect the fluorescence behavior of the CNCD, it was further optimized using fluorescent intensity as the objective [17]. The optimal mass fraction of CD was determined to be 0.10 wt%, as shown in Figure S2. The suspension was prepared and named CNCD, using the optimized experimental conditions.

### 2.5. Fabrication of CS/CNCD Composite Hydrogel

2 g of CS powder was dissolved in a beaker containing 98 g of 2 % acrylic acid solution (Aladdin, Shanghai, China). Subsequently, CNCD suspension (5 g) with different mass fractions (0.2 wt%, 0.4 wt%, 0.6 wt%, 0.8 wt%, and 1.0 wt%) was well dispersed into this solution through ultrasonic dispersion. GD (25 mL, 5 wt%) was then added. After 2 h of stirring, the mixture was heated to 60 °C, and was heat-preserved for 2 h. Since the mass ratio of CS and CNCD in the prepared hydrogel could affect its sorption behavior, it was further optimized with maximum adsorption capacity as the aim [18]. As shown in Figure S3, the optimal mass fraction of CNCD was identified to be 0.8 wt%. Using the optimized experimental conditions, the hydrogel was prepared. The obtained hydrogel was washed with water continuously to remove excess reactants before being cut into small discs, and labelled as CS/CNCD composite hydrogel for further testing. Furthermore, the blank hydrogel was created using the same method, with the exception that the CNCD was not introduced.

### 2.6. Batch Sorption Experiment

The sorption behaviors (effect of pH, temperature, contact time and initial concentration, and reusability) of the CS/CNCD composite hydrogel and the blank hydrogel were investigated by placing 3.0 g of these two adsorbents in different flasks containing 100 mL Cr(VI) solution, and shaking the flasks well. The pH was varied from 1 to 6, and the temperature varied in the range of 15 °C–45 °C. The effects of contact time (5–160 min) were tested. The concentration varied in the range of 20–120 mg/L. All of the experiments were averaged and repeated three times, with only the mean values displayed. The maximum deviation for duplicates was typically less than 5%.

### 2.7. Instruments

Scanning electron microscopy image was taken using a Quanta 250 FEG scanning electron microscope (FEI, Hillsboro, MI, USA). Fourier transform infrared spectra (FTIR) were collected via a NEXUS 870 spectrometer (Nicolet, Waltham, MA, USA). Fluorescence spectrum was obtained via a RF-5301PC luminescence spectrometer (Shimadzu, Kyoto, Japan). UV-visible spectrum was obtained via a 3100 UV-vis spectrophotometer (Shimadzu, Kyoto, Japan). X-ray photoelectron spectra (XPS) were measured using a PHI-5000 spectrometer (Ulvac-Phi, Kanagawa, Japan). X-ray diffraction (XRD) spectra were taken using a D8 ADVANCE diffractometer (Bruker AXS, Karlsruhe, Germany). Contact angle was determined using a Phoenix 300 contact angle analyzer (SEO Co. Ltd., Ansung, South Korea).

## 3. Results

### 3.1. Schematic Diagram

**Figure 1:**
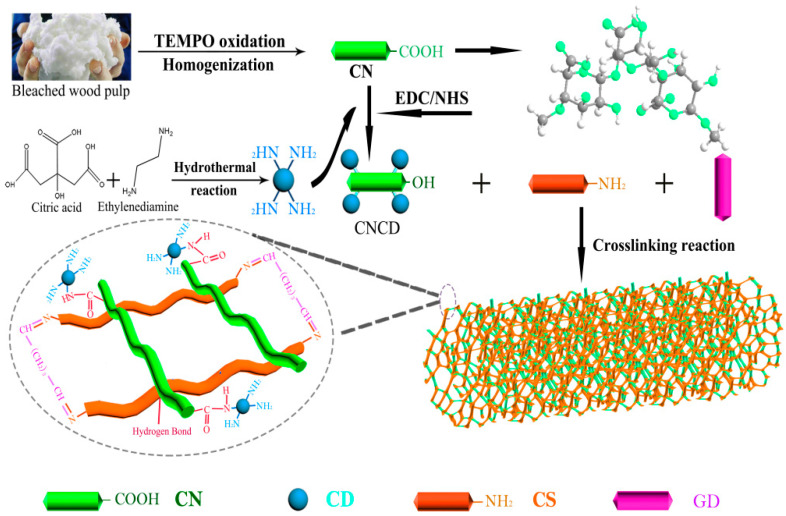
Schematic diagram of the synthesis process.

Firstly, we should re-examine our previous experiments. In this study, CN and CD were two additional elements, as shown in Figure 1. CNCD was first prepared by covalent bonding between CN and CD. Secondly, CNCD was combined with CS. Lastly, crosslinking took place in CS chains for constructing CS/CNCD composite hydrogel.

### 3.2. Characterization

**Figure 2:**
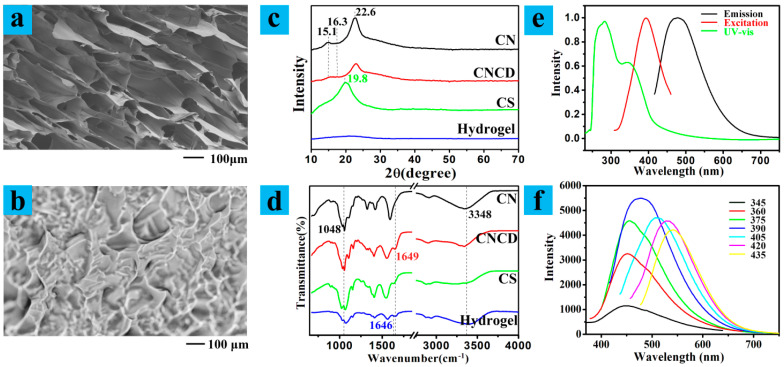
(**a**,**b**) SEM micrograph and optical microscope photo; (**c**) XRD patterns; (**d**) FTIR analysis; (**e**) optical properties; (**f**) emission spectra with varying excitation wavelengths.

Figure 2a,b show the microstructures of the CS/CNCD composite hydrogel. This hydrogel had a 3D porous structure, which improved swelling ability (swelling ratio of 129 g/g for deionized water at 25 °C). Using the crosslinking reaction, a 3D porous structure was required, and CD was wrapped [19]. Pore characteristics of the CS/CNCD composite hydrogel were evaluated in Figure S4, and the BET surface area and average pore width were 16.76 m^2^/g and 6.375 nm, respectively. Moreover, the contact angle of 17.7° showed the hydrophilicity of the CS/CNCD composite hydrogel [20]. 

The XRD pattern of CS, CN, CNCD, and CS/CNCD composite hydrogels is shown in Figure 2c. CN exhibited three peaks at 15.1°, 16.3°, and 22.6°, assigned to (101), (10$\bar{1}$), and (002) planes, respectively, indicating the crystallographic form of cellulose I [21]. These typical cellulose peaks were also observed in CNCD, indicating that the grafting of CD did not affect the crystalline structure of CN. CS had one broad peak at 19.8° corresponding to the amorphous structure [22]. In the pattern of CS/CNCD composite hydrogel, these peaks cannot be found clearly, since CS and GD produced a cross-linked porous structure, and CNCD were wrapped in this produced cross-linked porous structure [23].

The FTIR spectra of CS, CN, CNCD, and CS/CNCD composite hydrogels are shown in Figure 2d. The broad absorption band at approximately 3348 cm^−1^ was attributed to the O-H stretching vibration of CN, but in the cases of CS, CNCD, and CS/CNCD composite hydrogels, this band was also associated with the N-H stretching vibration. In addition, an obvious characteristic band (1048 cm^−1^) closely related to cellulose species was found in both CN and CNCD [24]. The appearance of an absorption band at 1649 cm^–1^ associated with –CO–NH– stretching vibrations confirmed that CD was grafted onto the CN [19]. Furthermore, the FTIR spectrum of CS/CNCD composite hydrogel contained several bands that differed from those of CS and CNCD. The broad absorption peak at 1646 cm^−1^ was specifically associated with –C=C–N=C– stretching vibrations, indicating that crosslinking occurred to construct a new structure containing –C=C–N=C– [25].

The optical properties of CS/CNCD composite hydrogel were investigated. The UV-vis spectrum exhibited two peaks at approximately 282 nm and 344 nm (Figure 2e), which could have originated from n–π* and π–π* transitions of CD [26]. The maximum emission peak of CS/CNCD composite hydrogel was centered at 478 nm under the 390 nm excitation. When λ_ex_ moved from 345 to 435 nm, the λ_em_ shifted from 449 to 541 nm (Figure 2f). The emissions of a CS/CNCD composite hydrogel with excitation-dependent properties were confirmed, which contributed to the surface state influencing the CD bandgap [27]. Moreover, the fluorescent stability of the CS/CNCD composite hydrogel was studied for different time periods, and the results are presented in Figure S5. The fluorescence and quantum yield (QY) were almost unchanged. Hence, this hydrogel had the potential for fluorescent probe.

### 3.3. Sorption of Cr(VI) 

**Figure 3:**
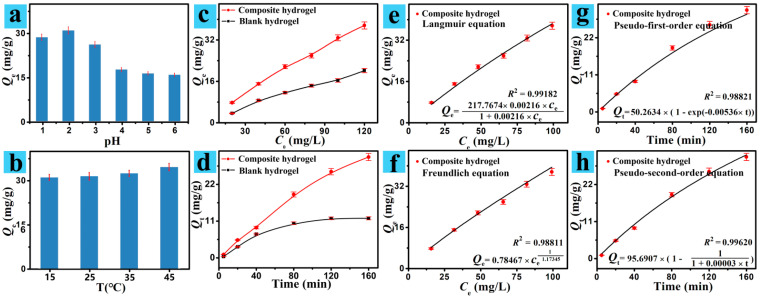
Effect of (**a**) pH and (**b**) temperature; (**c**,**d**) adsorption isotherm and sorption kinetic; (**e**,**f**) adsorption isotherms analyzed by two equations; (**g**,**h**) kinetics data analyzed by two equations.

The pH is a crucial parameter for Cr(VI) sorption. Hence, the sorption process is investigated at pH 1–6 (Figure 3a). As the pH was 1, the *Q*_e_ of the CS/CNCD composite hydrogel was low. According to Zeta potentials of the CS/CNCD composite hydrogel at different pHs (Figure S6), this hydrogel carried positive charges in the studied region. The Cr(VI) existed mainly as H_2_CrO_4_. Hence, there was the only weaker electrostatic attraction [28]. The second region was in the pH scope of 2–6, thus the *Q*_e_ of the CS/CNCD composite hydrogel began to decline. The Cr(VI) existed primarily as HCrO_4_ˉ, and this hydrogel was electrostatically attracted to HCrO_4_ˉ [29]. Due to the electrostatic attraction, this hydrogel had good adsorption performance for Cr(VI). As the pH increased in this region, the deprotonation reduced the *Q*_e_. As a result, the pH value 2 was chosen as the optimal pH.

As a major parameter during the adsorption process, the effects of temperature on the adsorption process of the CS/CNCD composite hydrogel towards Cr(VI) ion (Figure 3b) were explored. The *Q*_e_ of the CS/CNCD composite hydrogel increased, indicating that this sorption process may be endothermic [30]. It could be accounted for by the fact that the sorption capacity increases with increasing temperature. However, this increase was not so obvious in the temperature range of 15 °C–45 °C. Taking practical application into consideration, 25 °C was used for the subsequent experiments. 

The initial concentration of Cr(VI) ion can have an impact on the adsorption. As shown in Figure 3c, the *Q*_e_ of the CS/CNCD composite hydrogel and the blank hydrogel increased significantly with increasing concentration. Origin 7.0 software was used to perform least-squares curve fittings using the Langmuir and Freundlich equations. Fitting results are plotted in Figure 3e,f. The experimental data agreed well with the Langmuir equation with higher R^2^ (0.99182), showing this sorption onto the CS/CNCD composite hydrogel to be monolayer [31]. In addition, a maximum adsorption capacity (*Q*_m_) of 217.8 mg/g was obtained. Some researchers also created adsorbents for Cr(VI), the majority of which had lower *Q*_m_, as shown in Table 1.

Cr(VI) adsorption as a function of contact time was further observed (Figure 3d). Compared with the blank hydrogel, the adsorption capacities of the CS/CNCD composite hydrogel were high in the same amount of contact time. The pseudo-first-order kinetic equation and the pseudo-second-order kinetic equation are two common mathematical models used to describe the sorption kinetics of the CS/CNCD composite hydrogel. The least-squares curve fittings were performed by Origin 7.0 software with these two equations [40]. Fitting results are plotted in Figure 3g,h. The experimental data were better fitted with a pseudo-second-order equation with a higher R^2^ (0.99620), indicating that chemical sorption was controlling the sorption onto the CS/CNCD composite hydrogel.

Reusability is an important criterion for a material to be applicable in practical purposes. Hence, the adsorption-desorption cycles were taken using the eluent of HCl solution (1 mol/L); the results are shown in Figure S7. It was found that the CS/CNCD composite hydrogel retained 81.0% of its initial *Q*_e_ after four cycles. As a result, the CS/CNCD composite hydrogel could be reused and recycled.

### 3.4. Fluorescence Detection

**Figure 4:**
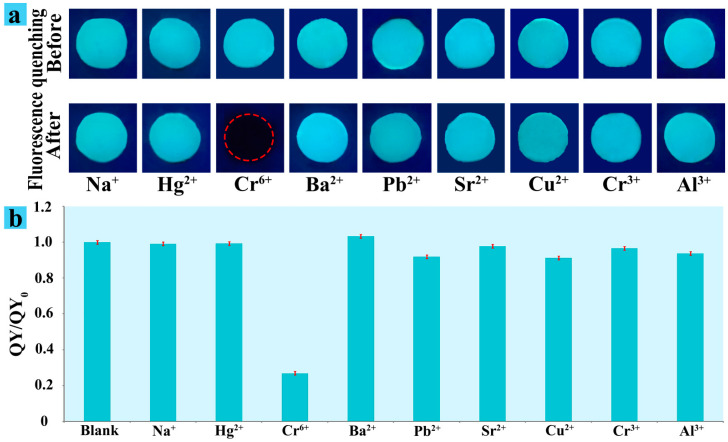
Fluorescence change (**a**) and QY (**b**) of CS/CNCD composite hydrogel with different heavy metals.

When the CS/CNCD composite hydrogel was challenged with coexisting metal ions (Na^+^, Hg^2+^, Cr^6+^, Ba^2+^, Pb^2+^, Sr^2+^, Cu^2+^, Cr^3+^, and Al^3+^), the selectivity, fluorescence change, and QY were investigated. As shown in Figure 4a,b, Cr(VI) could cause significant fluorescence quenching of the CS/CNCD composite hydrogel, whereas other metal ions caused only minor quenching. Moreover, a good selectivity of the CS/CNCD composite hydrogel towards Cr(VI) over other anions was observed in Figure S8. These results display the excellent selectivity of the CS/CNCD composite hydrogel towards Cr(VI).

**Figure 5:**
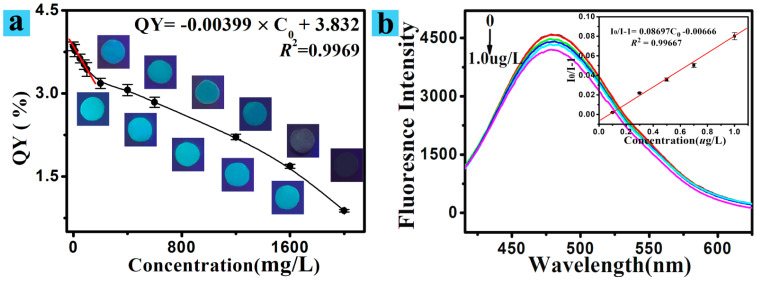
(**a**) Fluorescence change and QY of the CS/CNCD composite hydrogel mixed with different Cr(VI) concentrations; (**b**) fluorescent spectra of the CS/CNCD composite hydrogel mixed with lower Cr(VI) concentration, and the relationship between I_0_/I − 1 and concentration.

For the sensitivity study, various concentrations of Cr(VI) were thoroughly mixed with the CS/CNCD composite hydrogel. The fluorescence quenching decreased with Cr(VI) concentration, as shown in Figure 5a. The inset plot demonstrated a linear relationship between QY and Cr(VI) concentration (10–100 mg/L). Moreover, the fluorescence emission spectrum of the CS/CNCD composite hydrogel mixing with a lower concentration of Cr(VI) solution was collected, as shown in Figure 5b. As expected, the intensity of the signature of the peak at 478 nm decreased as concentration increased. The inset plot demonstrated a clear linear relationship between the intensity ratio (I_0_/I) − 1 and Cr(VI) concentrations (0.1–1.0 μg/L), with a detection limit of 0.04 μg/L. The analytical indices of this method (linear ranges of 0.1–1.0 μg/L and 10–100 mg/L, with a detection limit of 0.04 μg/L) were compared to those of the other reports. The comparison results listed in Table S1 clearly show that the CS/CNCD composite hydrogel had a lower detection limit.

The detection of Cr(VI) in tap and lake water was done by spiking different known concentrations of Cr(VI) solution to demonstrate the applicability of the CS/CNCD composite hydrogel. The average recoveries of Cr(VI) in spiked samples range from 99.17% to 101.01%, with a relative standard deviation less than 4.95%, as shown in Table S2. The results suggested that the CS/CNCD composite hydrogel had great potential for practical applications.

### 3.5. Cr(VI) Sorption and Detection Mechanism

**Figure 6:**
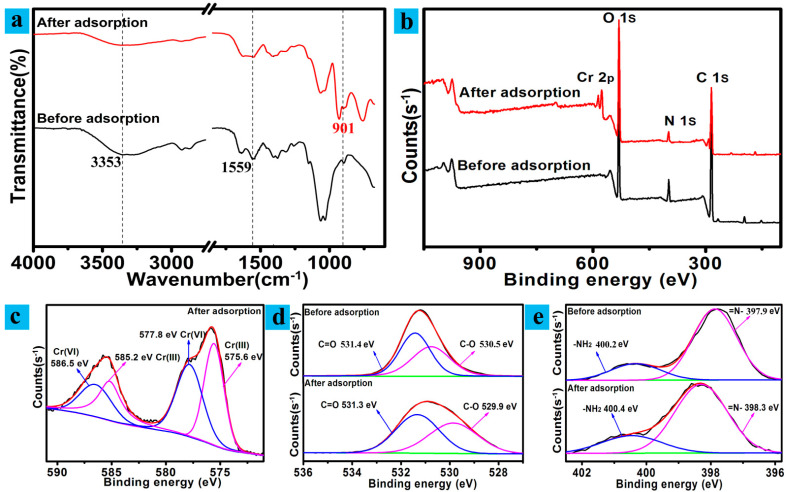
(**a**) FTIR spectra; (**b**) XPS total survey scan; (**c**–**e**) XPS spectra associated with Cr2p, O1s, and N1s.

The CS/CNCD composite hydrogel after adsorption processes was analyzed using FTIR in order to gain a better understanding of the sorption mechanism (Figure 6a). After sorption, the 3353 cm^−1^ peaks associated with N–H and O–H stretching vibrations and the 1559 cm^−1^ peak associated with –COO– stretching vibrations decreased. Furthermore, the 901 cm^−1^ peaks associated with Cr species increased [14]. It was indicated that the Cr(VI) sorption was closely related to hydroxyl, amino, and carboxyl groups.

To better understand the sorption mechanism, XPS analyses of the CS/CNCD composite hydrogel were performed. Total survey scans are displayed in Figure 6b. After sorption, four distinct peaks were observed: C1s (285 eV), N1s (400 eV), O1s (531 eV), and Cr2p (585 eV). This result confirmed that the CS/CNCD composite hydrogel adsorbed Cr(VI) ion. Figure 6c shows the high-resolution Cr2p spectra. It was shown that both Cr2p_3/2_ and Cr2p_1/2_ regions were split into two specific Cr(VI) and Cr(III) regions. The binding energies of 586.5 and 577.8 eV were related to Cr(VI), while the binding energies of 585.2 and 575.6 eV were related to Cr(III) [41]. The findings confirmed that a significant amount of Cr(VI) was reduced to Cr(III). Figure 6d shows the high-resolution XPS results of O1s. The O1s region was split into two components associated with C=O and C–O. The integral area ratio of these two components grew, indicating that some C–O groups could be oxidized into C=O groups. Peaks at 398.3 and 400.4 eV were observed in the high-resolution N1s spectrum (Figure 6e), which were related to imine (=N–) and amine (–NH_2_) groups. The intensity of the signature from –NH_2_ was observed to rise after sorption, which indicated that the amino group participated in the sorption. 

To elucidate the detection mechanism, UV-vis and fluorescence excitation spectra were obtained. The UV-Vis band of Cr(VI) overlapped with the CS/CNCD composite hydrogel (Figure S9a), implying that Cr(VI) detection could be due to the IFE. As shown in Figure S9b–d, the average fluorescence life of the CS/CNCD composite hydrogel (τ* = 5.9 ns) was very close to that of the CS/CNCD composite hydrogel adsorbed with Cr(VI) (τ* = 6.0 ns), confirming that there was static quenching. The IFE and static quenching were used to power the detection mechanism.

**Figure 7:**
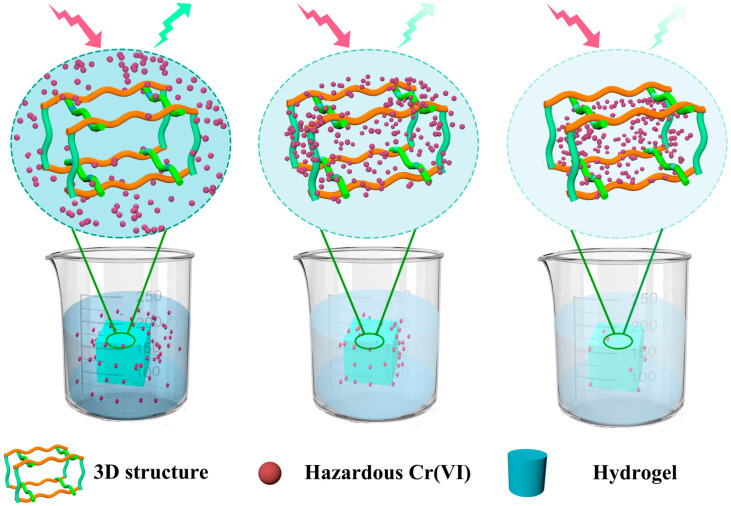
Sorption and detection mechanism of the CS/CNCD composite hydrogel.

According to the above-mentioned analytical results, the CS/CNCD composite hydrogel’s Cr(VI) sorption and detection mechanism are as follows (Figure 7). The Cr(VI) was first enriched into the interface via electric attraction. After that, Cr(VI) was gradually diffused to the inside along 3D porous structures. In this diffusion process, a considerable amount of Cr(VI) was reduced to Cr(III), then some of the Cr(III) ions interacted with the hydroxyl and carboxyl groups. Due to these specific structures, the CS/CNCD composite hydrogel had a high adsorption capacity. Concurrently, Cr(VI) was enriched into the CD via electric attraction, resulting in fluorescence quenching. As a result, the CS/CNCD composite hydrogel possessed sensitive detection capability.

### 3.6. Economic Feasibility

The production cost is a critical factor in considering whether an adsorbent has potential. The net cost of producing an adsorbent typically includes the cost of raw material, reaction reagent, and operating reaction equipment [42]. Table S3 shows the total cost for preparing 1 kg of the CS/CNCD composite hydrogel. In comparison with the commercially activated carbon (AC), the CS/CNCD composite hydrogel was cheaper. Moreover, Table S4 shows the cost incurred in adsorbing 1 g Cr(VI) using the CS/CNCD composite hydrogel compared to AC. According to this cost analysis, the cost of removing 1 g Cr(VI) for the CS/CNCD composite hydrogel was only CNY 0.62, which was nearly 58.8 times less expensive than AC (CNY 36.48). The CS/CNCD composite hydrogel appears to be a promising adsorbent for Cr(VI) removal.

## 4. Conclusions

In this study, a novel hydrogel was prepared via CS incorporating CN modified with CD, followed by crosslinking with GD with a strong sorption ability and sensitive detection ability for Cr(VI). The effects of various parameters on Cr(VI) sorption were investigated, including initial pH, temperature, contact time, and initial concentration. The CS/CNCD composite hydrogel had high absorption properties, with a maximum adsorption capacity of 217.8 mg/g. Meanwhile, this hydrogel was employed for selective and quantitative detection of Cr(VI) with linear ranges of 0.1–1.0 μg/L and 10–100 mg/L, and a detection limit of 0.04 μg/L. Furthermore, an economic analysis of this hydrogel was performed, which revealed that the cost of removing 1 g Cr(VI) for the CS/CNCD composite hydrogel was nearly 58.8 times lower than that of AC. All of this demonstrated that the developed hydrogel could be used to treat Cr(VI) wastewater.

## Figures and Tables

**Table 1:** Maximum sorption capacities in comparison with previous work.

Sample	*Heavy Metals*	*Q*_m_ (mg/g)	References
Tetraethylenepentamine crosslinked chitosan oligosaccharide hydrogel	*Cr(VI)*	150.5	[32]
Chitosan-based hydrogel	*Cr(VI)*	93.0	[33]
Fe_3_O_4_ NPs/CS/glyoxal hydrogel	*Cr(VI)*	27.3	[34]
Activated carbon	*Cr(VI)*	5.46	[35]
Magnetic chitosan/PVA hydrogel	*Cr(VI)*	2.2	[36]
Chitosan composite hydrogel	*Cr(VI)*	66.9	[37]
Chitosan/montmorillonite composite hydrogel	*Cr(VI)*	78.0	[38]
HPAM-chitosan gel bead	*Cr(VI)*	66.1	[39]
The CS/CNCD composite hydrogel	*Cr(VI)*	217.8	This work

## Data Availability

All the data are available within the manuscript.

## Supplementary Materials

The following are available online at https://www.mdpi.com/article/10.3390/polym13213788/s1, Figure S1: TEM image of the CDs, Figure S2: Fluorescence spectra (a) and fluorescence intensity (b) of a series of CNCD prepared by using different concentrations of CD solution, Figure S3: Maximum adsorption capacities for Cr(VI) of a series of hydrogels, Figure S4: Pore characteristics of the CS/CNCD composite hydrogel, Figure S5: Fluorescent stability of the CS/CNCD composite hydrogel, Figure S6: Zeta potentials of the CS/CNCD composite hydrogel, Figure S7: Adsorption–desorption cycles, Figure S8: Fluorescence change and QY of CS/CNCD composite hydrogel with different anions and Cr(VI), Figure S9: (a) UV-Vis absorption spectrum of Cr(VI) and excitation spectrum of the CS/CNCD composite hydrogel. (b) Fluorescence decay curves of the CS/CNCD composite hydrogel and the CS/CNCD composite hydrogel with adsorbed Cr(VI) ion. (c,d) Fluorescence decay curves fitted by second-order exponential functions, Table S1: Comparison of sensing performance of different sensors for Cr(VI) detection, Table S2: Recovery and RSD of Cr(VI) in tap and lake water samples, Table S3: Breakup and total cost for preparing 1 kg of CS/CNCD composite hydrogel, Table S4: Cost of adsorbent for the removal of 1 g of Cr(VI).
