# Supplementary material for: Effective Adsorption and Sensitive Detection of Cr(VI) by Chitosan/Cellulose Nanocrystals Grafted with Carbon Dots Composite Hydrogel
Source: Polymers (Basel). 2021 Nov 1;13(21):3788. doi: 10.3390/polym13213788 (PMC8588005; doi:10.3390/polym13213788)
Supplement: Supplementary file 1 [file polymers-13-03788-s001.zip › polymers-1434992-supplementary.pdf]

Supplementary Material

Effective Adsorption and Sensitive Detection of Cr(VI) by Chitosan/Cellulose Nanocrystals Grafted with Carbon Dots Composite Hydrogel

Hua Zeng [1], Zhiyuan Hu [1], Chang Peng [2], Lei Deng [2] and Suchun Liu [1,*]

1 College of Food Science and Technology, Hunan Agricultural University, Changsha 410128, China

2 School of Chemistry and Materials Science, Hunan Agricultural University, Changsha 410128, China

* Correspondence: hunaulsc@163.com

Contents

1. **Figure S1.** TEM image of the CDs.

2. **Figure S2.** Fluorescence spectra (a) and fluorescence intensity (b) of a series of CNCD prepared by using different concentrations of CD solution.

3. **Figure S3.** Maximum adsorption capacities for Cr(VI) of a series of hydrogels

4. **Figure S4.** Pore characteristics of the CS/CNCD composite hydrogel.

5. **Figure S5.** Fluorescent stability of the CS/CNCD composite hydrogel.

6. **Figure S6.** Zeta potentials of the CS/CNCD composite hydrogel.

7. **Figure S7.** Adsorption–desorption cycles.

8. **Figure S8.** Fluorescence change and QY of CS/CNCD composite hydrogel with different anions and Cr(VI).

9. **Figure S9.** (a) UV–vis absorption spectrum of Cr(VI) and excitation spectrum of the CS/CNCD composite hydrogel. (b) Fluorescence decay curves of the CS/CNCD composite hydrogel and the CS/CNCD composite hydrogel with adsorbed Cr(VI) ion. (c–d) Fluorescence decay curves fitted by second-order exponential functions.

10. **Table S1.** Comparison of sensing performance of different fluorescent sensors for Cr(VI) detection.

11. **Table S2.** Recovery and RSD of Cr(VI) in tap and lake water samples.

12. **Table S3.** Breakup and total cost for preparing 1 kg of CS/CNCD composite hydrogel.

13. **Table S4.** Cost of adsorbent for the removal of 1 g of Cr(VI).

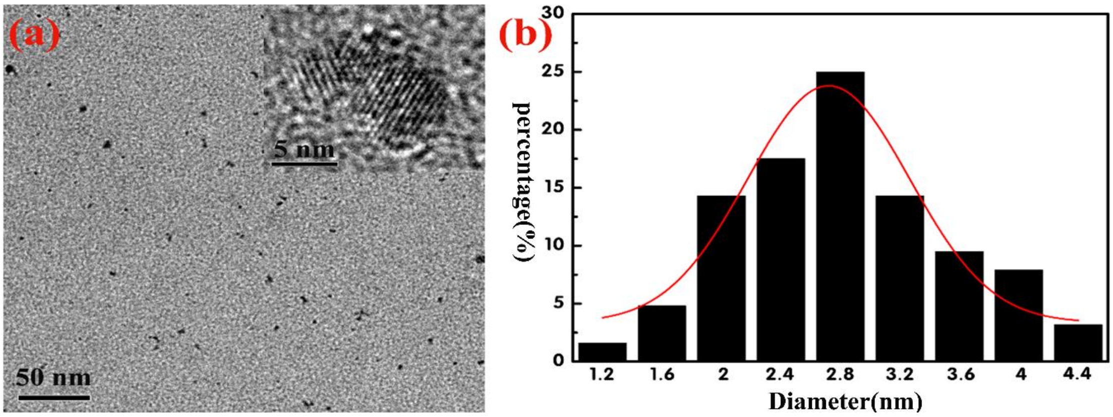

Figure S1. TEM image of the CDs.

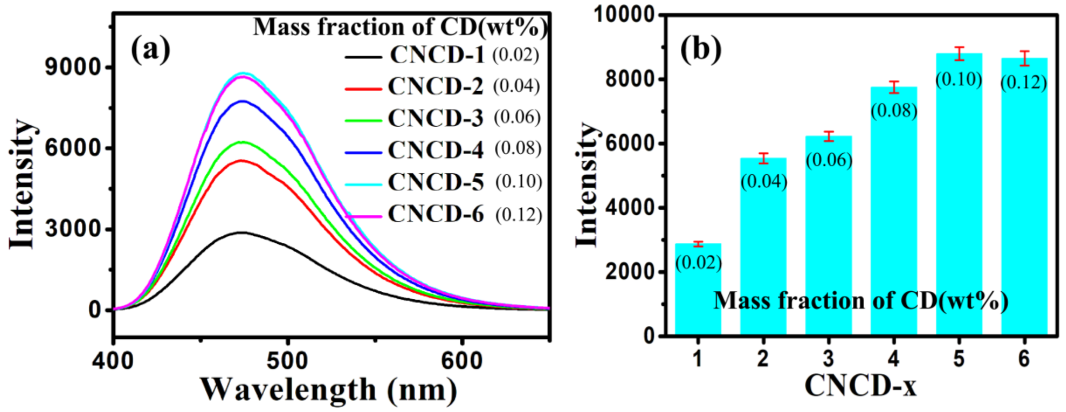

Figure S2. Fluorescence spectra (a) and fluorescence intensity (b) of a series of CNCD prepared by using different concentrations of CD solution.

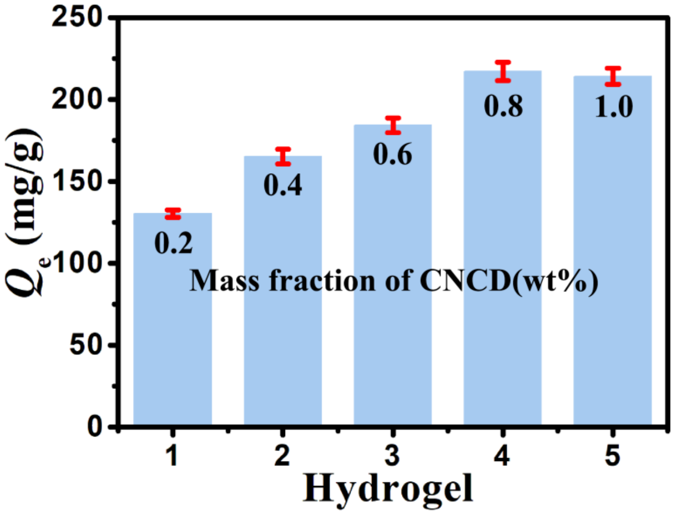

Figure S3. Maximum adsorption capacities for Cr(VI) of a series of hydrogels.

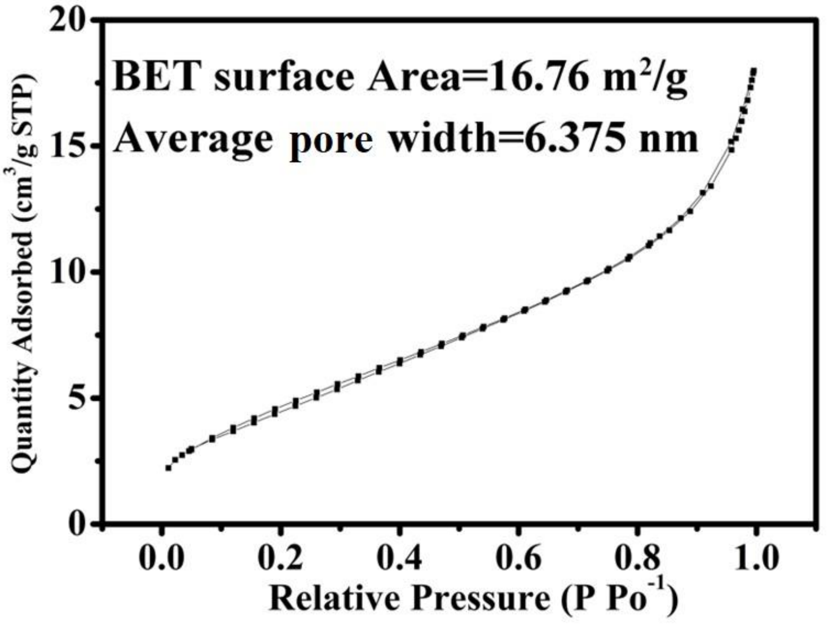

Figure S4. Pore characteristics of the CS/CNCD composite hydrogel.

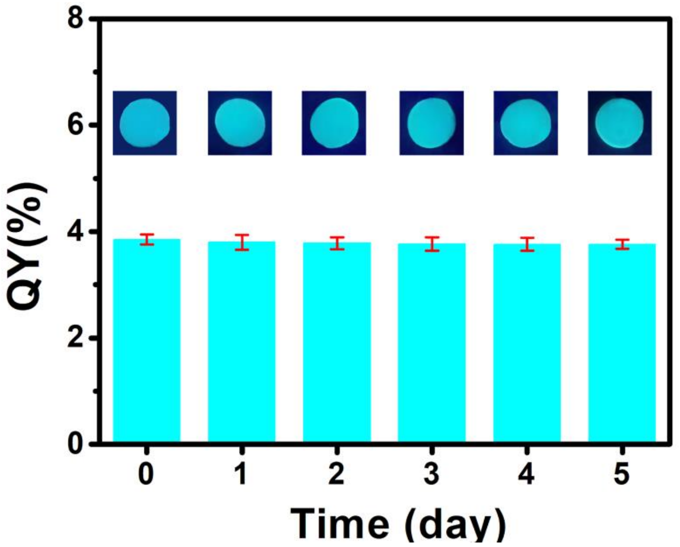

Figure S5. Fluorescent stability of the CS/CNCD composite hydrogel.

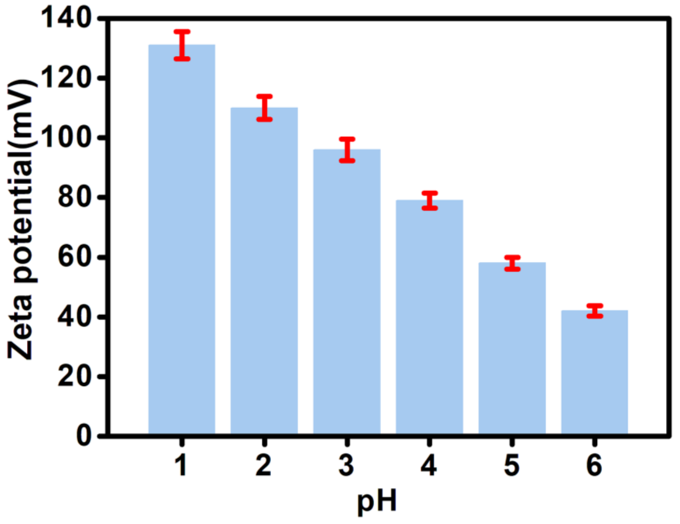

Figure S6. Zeta potentials of the CS/CNCD composite hydrogel.

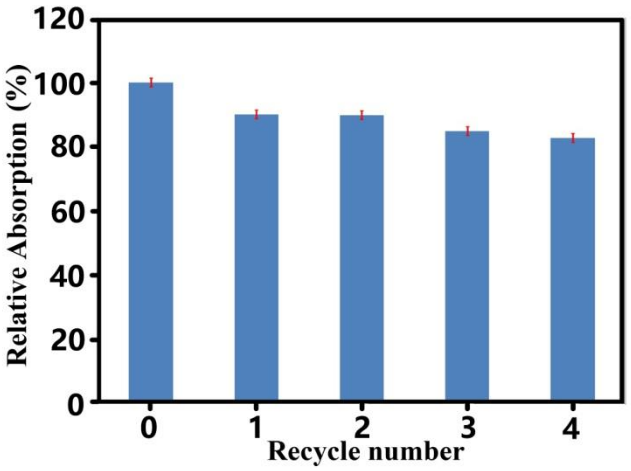

Figure S7. Adsorption–desorption cycles.

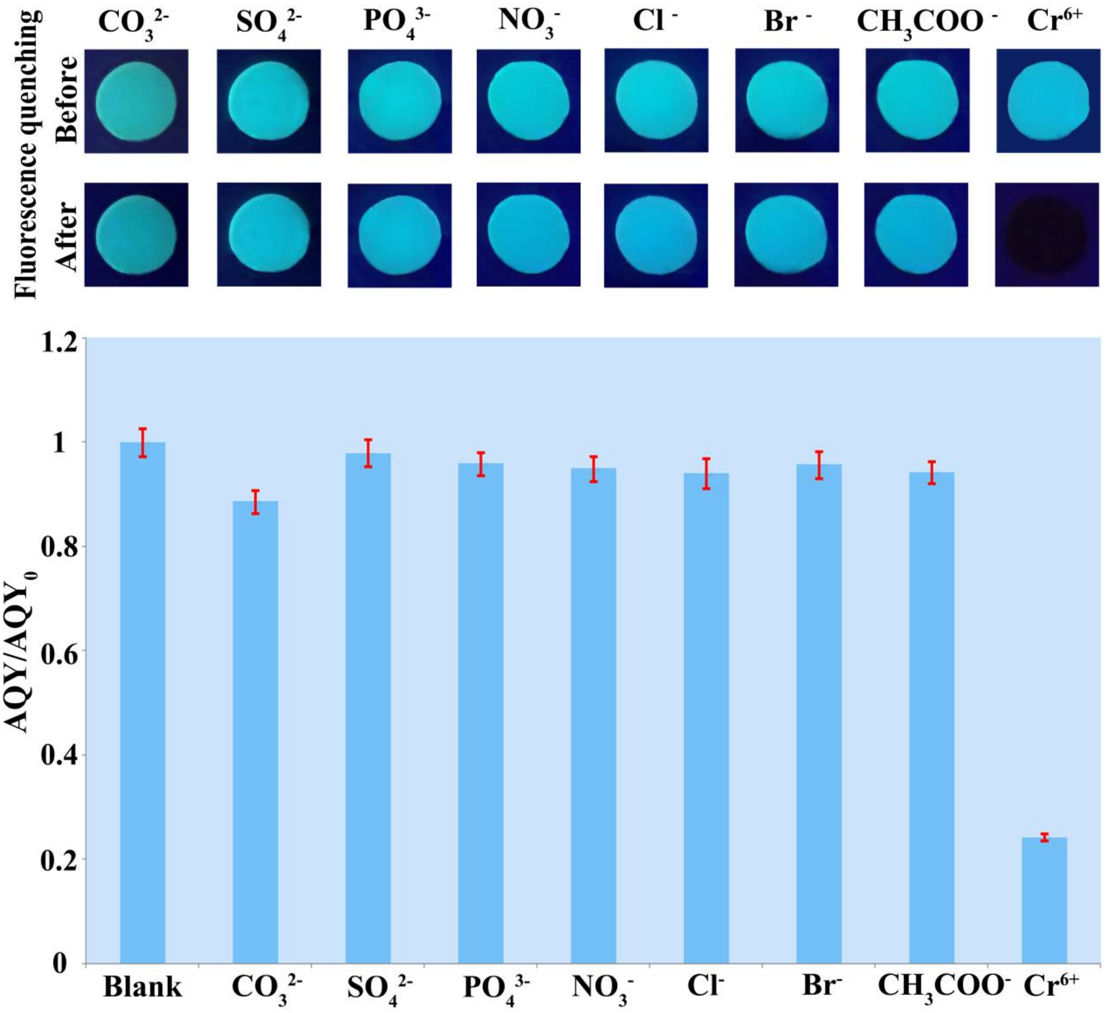

Figure S8. Fluorescence change and QY of CS/CNCD composite hydrogel with different anions and Cr(VI).

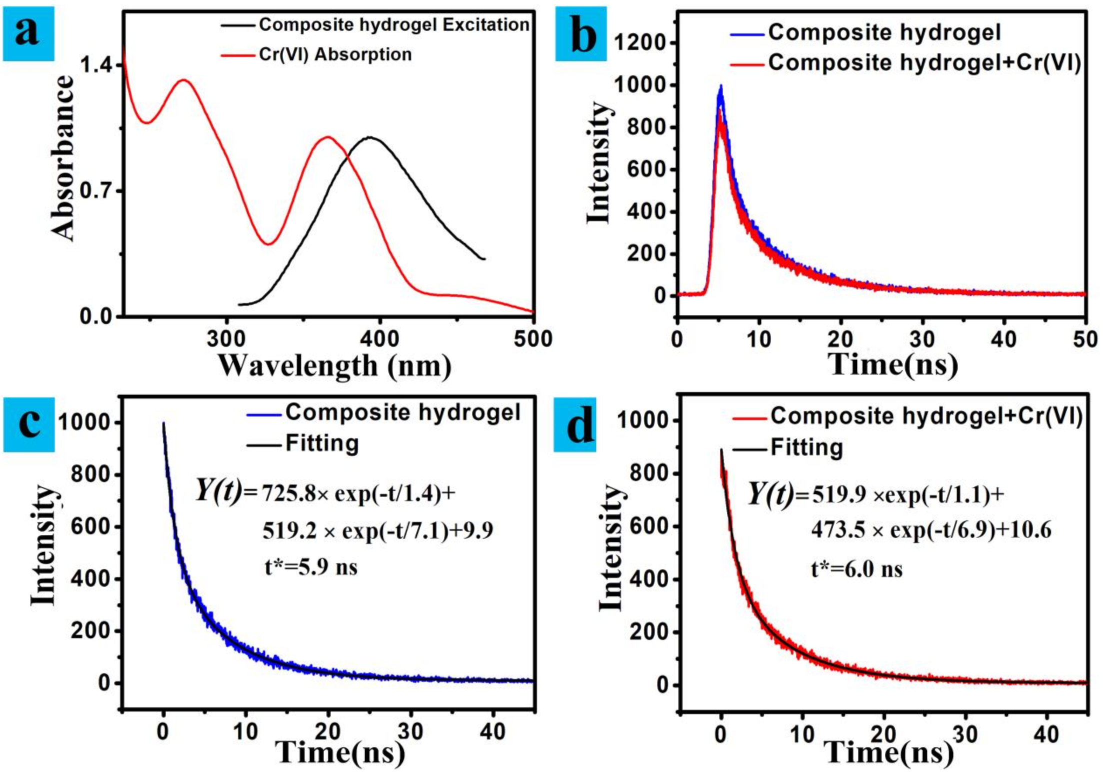

Figure S9. (a) UV–vis absorption spectrum of Cr(VI) and excitation spectrum of the CS/CNCD composite hydrogel. (b) Fluorescence decay curves of the CS/CNCD composite hydrogel and the CS/CNCD composite hydrogel with adsorbed Cr(VI) ion. (c–d) Fluorescence decay curves fitted by second-order exponential functions.

Table S1. Comparison of sensing performance of different fluorescent sensors for Cr(VI) detection.

Fluorescent probe	Linear range (mg/L)	Detection limit (mg/L)	Reference
CDs from citric acid and acrylamide	0.104-9.36	0.109	[1]
CDs from Tulsi leaves	0.083-2.6	0.005	[2]
CDs from citric acid and cystamine dihydrochloride	0.052-4.16	0.045	[3]
CDs from citric acid and N,N′-bis(2-aminoethyl)-1,2-ethanediamine	4.16-52	7.28	[4]
CDs from citric acid and glycine	0.26-10.4	0.216	[5]
CDs from 1-(2-Pyridylazo)-2-naphthol and cobalt chloride	0.26-6.5	0.061	[6]
S and N dual-doped carbon dots	0.00338-0.010296<br>1.16-157	0.00003	[7]
CS/CNCD composite hydrogel	0.0001-0.001<br>10-100	0.00004	In this paper

Table S2. Recoveries and relative standard deviations of Cr(VI) in tap and lake water samples.

Sample	Added (mg/L)	Found (mg/L)	Recovery (%)	RSD (%)
Tap water	0	ND	ND	ND
	10	9.96	99.57	2.62
	50	50.08	100.15	0.33
	100	100.06	100.06	0.14
	200	200.03	101.01	0.04
Lake water	0	ND	ND	ND
	10	9.92	99.17	4.95
	50	50.09	100.18	0.68
	100	99.94	99.94	0.25
	200	200.05	100.03	0.10

ND: not detected

Table S3. Breakup and total cost for preparing 1 kg of CS/CNCD composite hydrogel.

No.	Material	Unit Cost(CNY)	Amount used	Net Price(CNY)
1	Bleached Wood pulp	6.1/1000g	16	0.10
2	TEMPO	63/25g	0.32	0.81
3	Sodium bromide	67/500g	1.6	0.21
4	NaClO	17/500ML	48	1.63
5	Cost of homogenization	0.8/1KWH	2000W	1.60
6	Citric acid	46/500g	1	0.09
7	Ethylenediamine	119/25mL	128uL	0.61
8	Cost of heating 1	0.8/1KWH	500W*5	2.00
9	EDC	228/100g	2.6	5.93
10	NHS	202/100g	2.1	4.24
11	AA	14.8/500ML	593	17.55
12	CS	155/1000g	606	93.93
13	GD	27/2.5L	378	4.08
14	Cost of heating 2	0.8/1KWH	500W*4	1.60
15	Total Cost (CNY)			134.38

Table S4. Cost of adsorbent for the removal of 1 g of Cr(VI).

No.	Adsorbent	Adsorption capacity(mg/g)	Cost of Adsorbent (CNY/kg)	Cost of adsorbent for removal of 1 g of Cr(VI) (CNY)
1	The CS/CNCD composite hydrogel	217.80	134.38	0.62
2	Activated carbon	5.46	199.00	36.48

References

[1] C. Li, W. Liu, X. Sun, W. Pan, J. Wang, Multi sensing functions integrated into one carbon-dot based platform via different types of mechanisms, Sensor. Actuat. B-Chem. 252 (2017) 544-553.

[2] S. Bhatt, M. Bhatt, A. Kumar, G. Vyas, T. Gajaria, P. Paul, Green route for synthesis of multifunctional fluorescent carbon dots from Tulsi leaves and its application as Cr(VI) sensors, bio-imaging and patterning agents, Colloid. Surface. B 167 (2018) 126-133.

[3] J. Chen, J. Liu, J. Li, L. Xu, Y. Qiao, One-pot synthesis of nitrogen and sulfur co-doped carbon dots and its application for sensor and multicolor cellular imaging, J. Colloid Interf. Sci. 485 (2017) 167-174.

[4] K. Lu, J. Lin, C. Lin, C. Chen, Y. Yeh, A fluorometric paper test for chromium(VI) based on the use of N-doped carbon dots, Microchim. Acta 186 (2019) 227.

[5] H. Wang, S. Liu, Y. Xie, J. Bi, Y. Li, Y. Song, S. Cheng, D. Li, M. Tan, Facile one-step synthesis of highly luminescent N-doped carbon dots as an efficient fluorescent probe for chromium(VI) detection based on the inner filter effect, New J. Chem. 42 (2018) 3729-3735.

[6] H. Zhang, Y. Wang, S. Xiao, H. Wang, J. Wang, L. Feng, Rapid detection of Cr(VI) ions based on cobalt(II)-doped carbon dots, Biosens. Bioelectron. 87 (2017) 46-52.

[7] S. Song, F. Liang, M. Li, F. Du, W. Dong, X. Gong, S. Shuang, C. Dong, A label-free nano-probe for sequential and quantitative determination of Cr(VI) and ascorbic acid in real samples based on S and N dual-doped carbon dots, Spectrochim. Acta a. 215 (2019) 58-68.
